# Computed tomography: a beneficial diagnostic tool for the evaluation of the canine prostate?

**DOI:** 10.1186/s12917-017-1016-5

**Published:** 2017-05-08

**Authors:** N.S.M. Kuhnt, L. K. Harder, I. Nolte, P. Wefstaedt

**Affiliations:** Small Animal Clinic, University of Veterinary Medicine Hannover, Foundation, Hannover, Bünteweg 9, D-30559 Hannover, Germany

**Keywords:** prostate gland, computed tomography, dog, Hounsfield Unit

## Abstract

**Background:**

Prostatic diseases in intact male dogs are common. However, studies about the computed tomographic (CT) examination of the prostate in dogs are rare. The aim of the present study was to evaluate age related-changes in the canine prostate with the help of the CT and to evaluate whether measuring Hounsfield Units (HUs) in different morphological conditions of the prostate is of diagnostic value.

Fifty pre- and post-contrast CT scans of the prostate of dogs were evaluated and divided into three groups according to the tissue structure: Group1 dogs with homogenous prostate tissue (16/50); group 2 with prostate cysts (26/50) and group 3 with inhomogeneous prostate tissue (8/50). The prostatic dimensions were measured and the ratio between length, height and width and the sixth lumbar vertebra was calculated. Median values of prostatic attenuation measured in HUs, using regions of interests (ROIs) were determined on pre- and post- contrast scans over the whole length of the prostate. The results were compared to the dog’s age.

Furthermore, the CT Images were compared with the results of ultrasonography (47/50).

**Results:**

On pre-contrast scans HUs within ROIs placed in the prostate did not differ statistically significantly between the different morphological groups (1: 37.7; 2: 36.3; 3: 39.8 HU). HUs within on the post- contrast scans showed statistically significant differences between the groups. Group one had a mean density of 93.6 HU, group two had a mean density of 106.1 HU and group three had one of 138.2 HU. The prostatic size in the first group was smaller than in the other groups, whereas the largest prostates were found in the second group.

In six cases the post-contrast CT scan showed results that differed from the ultrasound examination. Dogs had a homogenous tissue in ultrasonography while the CT scan revealed an inhomogeneous tissue structure.

**Conclusions:**

The CT examination can be a beneficial diagnostic tool for examining the prostatic size and for evaluating the prostatic tissue. The different HUs reflected age-related changes and alterations in the prostate while measuring the density of the prostate. Contrast agent application enables a more specific analysis of the prostate to be carried out and for precise changes in tissue structure to be observed.

## Background

The prostate gland produces the fluid for the transportation and nutrition of sperm and is the only accessory sexual gland of male dogs [1]. Pathological changes in the prostate in older intact male dogs (>6 years) are a common reason for consulting a veterinarian [2]. The incidence for prostatic disorders in 72,300 male dogs amounted to 0.7%. Benign prostatic hyperplasia (BPH) was the most frequently observed prostatic disease in dogs, followed by prostatitis (38.5%) and abscesses (7.7%) [3]. While BPH is not found in beagles younger than 2 years, the prevalence for BPH is 100% in beagles older than 6 years [4]. In the study of Black et al. in a sample of 85 dogs, prostatic cysts could be found with a prevalence of 14% in dogs older than 3 years [5] and can develop of a BPH [6] or be of congenital origin [7].

For an effective therapy and prognosis of prostatic disorders a proper diagnosis is indispensable [8]. As rectal palpation and x-ray examination may not be a sufficient tool for examining the prostate structure, further diagnostic measures including imaging are needed [9]. Transabdominal ultrasonography of the prostate enables a visualization of the prostate’s structure [2], but may be limited by the partially intrapelvic position of the gland. A transrectal ultrasound is recommended to achieve images of the intrapelvic parts of the prostate gland [10]. Due to the need for sedation [11] it is not commonly applied for prostatic imaging in canine practice [12].

In general, CT imaging of abdominal organs has several benefits: organs can be imaged without superimposition; due to the high resolution small structures can be detected and thanks to image reconstruction organ size and shape can be evaluated in several planes [13–16]. Accordingly, CT examination is considered as a helpful tool for evaluating the canine prostate gland [17]. However, only few studies have used CT to further investigate its diagnostic benefits for examining the prostate in dogs [15, 18].

In the study of Lee et al. [15] morphological features of the prostate gland in CT examination of 35 adult intact male-dogs were examined, and several changes were seen. The CT measurement of the prostatic size showed similar values as those measured by sonography and patho-morphology [15]

Developing this CT imaging approach further, Pasikowska et al. [18] examined the prostatic size, the ratio of the height, length and width and attenuation values of the prostate in 40 dogs according to the prostate health status. The attenuation values of the prostate tissue in dogs with BPH were lower in the pre- and post-contrast images than in the healthy dogs. Furthermore, the prostatic dimensions in dogs with a BPH were higher than in healthy dogs.

The aim of the present study was to evaluate the relationship of the dog’s age with the CT findings of prostate morphology, including size and attenuation of the prostate gland, in pre- and post-contrast scans. Furthermore, the study compares the structural changes seen in CT-images with the results of sonographic examinations. Additionally, the tissue surrounding cystic structures were characterized in order to find differences to normal homogenous prostatic tissue.

## Methods

### Patients

CT-records of client-owned dogs that were presented at the Small Animal Clinic, University of Veterinary Medicine Hannover, Foundation for diagnostic imaging between October 2007 and June 2016, were evaluated in this retrospective study. CT-records were included in the study when the patient was an intact male dog and non-contrast and contrast CT scans of the prostate gland were performed. Exclusion criteria were imaging artifacts on CT records of the prostate gland, like metal streak artifacts from an orthopedic implant. Another exclusion criterion was a missing 6th lumbar vertebra in the image stack.

50 intact male dogs of various breeds met the inclusion criteria. The patient’s age, breed and in most cases also the dog’s weight and nutritional condition were included as additional information.

The dogs’ age range was between 8 months to 14 years (mean 7.5 years) and their body weight ranged between 5.5 kg and 49 kg (mean 26 kg).

The reasons for the CT examinations varied (Table 1).

**Table 1:** Overview of the reasons for CT examination

Primary indication of CT examination	Number of dogs
● Abdominal mass	7
● Anal mass ● Mass on hind limbs ● Intestinal masses ● Prostatic symptoms (Tenesmus, hematuria)	4 4 4 4
● Insulinomas ● Abdominal foreign body	3 3
● Ectopic ureter ● Perineal hernia ● Vestibular syndrome ● Hepatopathy ● Vomitus	2 2 2 2 2
● Intervertebral disc disease ● Ascites ● Anemia ● Spondylosis ● Kidney tumor ● Cystitis ● Abdominal pain ● Back mass ● Thyroid mass ● Epilepsy ● Orthopedic disease	1 1 1 1 1 1 1 1 1 1 1

### CT examination

CT scans were performed at the Small Animal Clinic of the University of Veterinary Medicine Hannover, Foundation with a 64 multi-detector-row CT scanner (Phillips Brilliance 64, Philips GmbH, Hamburg, Germany) with the dogs being in dorsal or ventral recumbency during examination.

The anesthesia was induced with levomethadone (L-Polamivet 0.2 mg/kg; CP-Pharma Handelsgesellschaft mbH, Burgdorf, Germany), diazepam (Ziapam®,0.5 mg/kg, Laboratoire TVM, Lempdes, France) and propofol (dose according to effect; Narcofol® CP-Pharma Handelsgesellschaft mbH, Burgdorf, Germany). During the CT examination inhalation anesthesia was maintained with isoflurane (Isofluran CP®, CP-Pharma Handelsgesellschaft mbH, Burgdorf, Germany). Due to the different weight of the patients CT-examination scan parameters (current/voltage/slice thickness/pitch) varied between the dogs. For dogs with a weight under 20 kg a CT imaging protocol with the following parameter was used: voltage 120 kV, current: 30 mAS. Dogs weighing more than 20 kg were scanned with a higher current of 200 mAS and a voltage of 120 kV. In both cases a slice thickness of 2 mm and a pitch of 1.171 were used. Within each dog scan parameters were kept constant for pre- and post-contrast examination. The contrast scan was performed using the same scanning parameters as mentioned above using a non-ionic iodinated contrast agent (Xenetix® 300, Guerbet GmbH; Sulzbach, Germany) at a dose of 2 ml/kg bodyweight administered via a bolus tracking pump into the vena cephalica antebrachii or vena saphena lateralis. In every examination the local tracker was positioned in the aorta. Independently of a patient’s weight the post-contrast scan started 49 s after reaching a HU of 150. The field of view included the whole prostate and the sixth lumbar vertebra.

### CT image analysis

The CT-images were stored in DICOM format and reconstruction was made using an image-processing workstation (Extended Brilliance Workspace, Philips Medical Systems, Ohio,USA) and the program ImageJ (Wayne Rasband, National Institutes of Health, Behesda, MD, USA).

**Fig.1:**
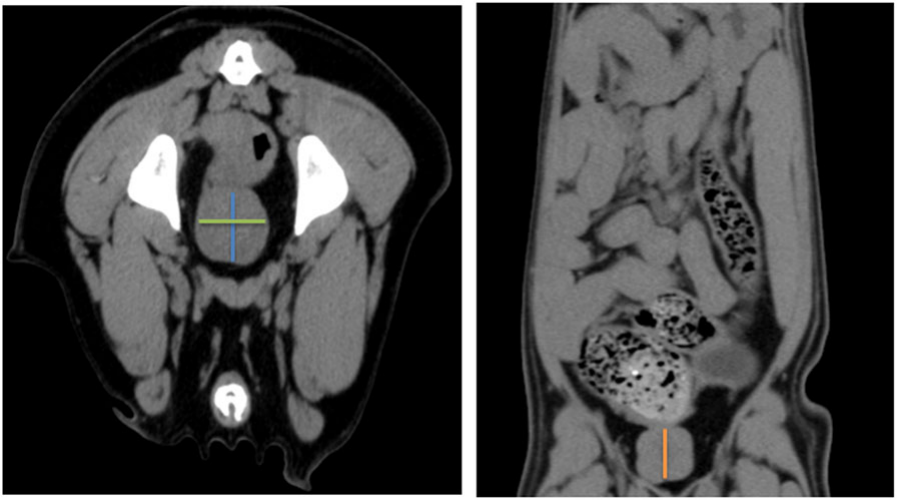
Overview of prostatic size measurements on a transverse image (left): The blue line represents the height, the green line the width of the prostate (intact male dog, German shepherd, 2 years old). On the dorsal plane (right): orange line represents the length (intact male dog, German long-haired, 2 years old).

All CT data sets were assessed in transverse planes and reconstructed in sagittal and dorsal planes. For the following analysis all CT image stacks were intersected with 2 mm slice thickness. Measurement of the prostatic size was carried out according to the study of Lee [15]. Therefore, the prostate’s width and height were measured on transverse images. Regarding the whole image stack, the sectional image showing the highest diameter of the prostate was chosen for measuring the prostate width and height. For this measurement a vertical and a horizontal line was drawn through the intraprostatic part of the urethra (Fig. 1). The dorsal plane was chosen for measuring the prostate’s length.

**Fig. 2.**
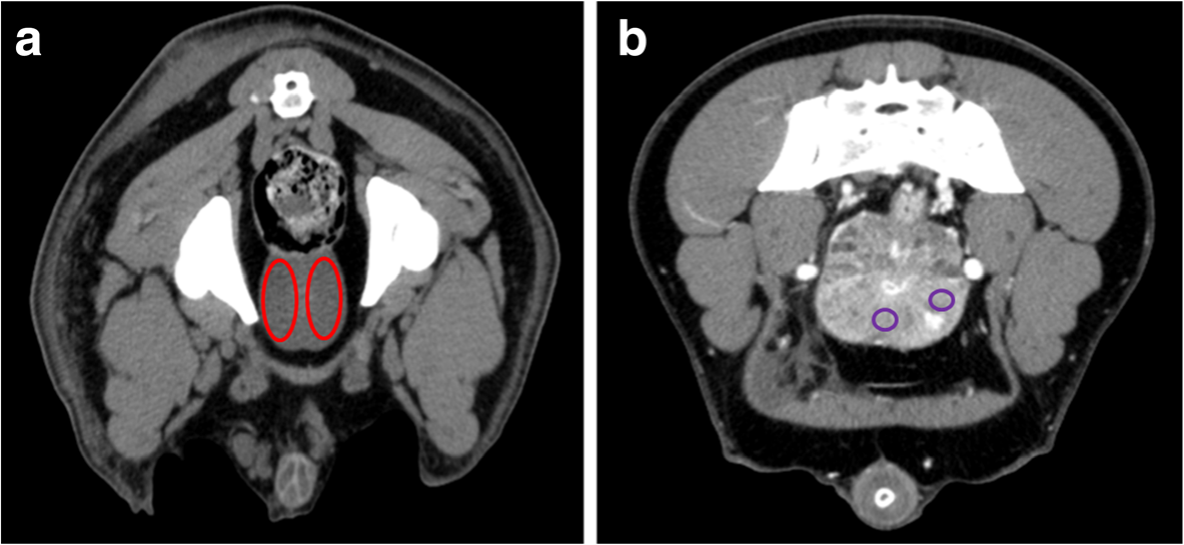
CT images of the prostate gland in transverse plane. **a** ROIs were drawn separately in the right and left lobe of the prostate. (Intact male dog, Hovawart, 2 years old) **b** Additionally, smaller ROIs for measuring the HU in the surrounding tissue of cysts. (Intact male dog, Doberman, 4 years old)

**Fig. 3:**
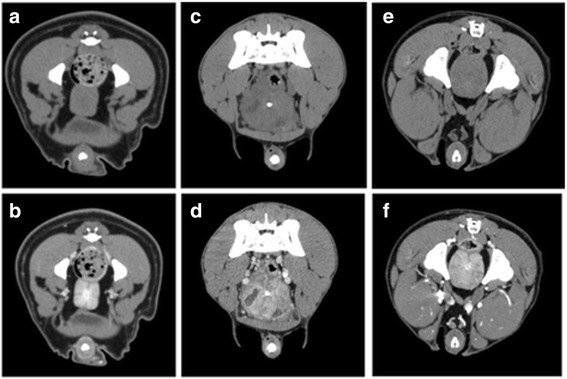
Overview of the three morphological groups: **a**) a homogenous prostate displayed in a pre-contrast scan (intact male dog, pug, 1 year old) **b**) a homogenous prostate in a post-contrast scan **c**) a prostate with cysts in a pre-contrast scan (intact male dog, mixed breed, 9 years old) **d**) a prostate with cysts displayed in a post- contrast scan **e**) an inhomogenous prostate displayed in a precontrast scan (intact male dog, Briard, 3 years old) **f**) an inhomogenous prostate in a postcontrast scan

**Fig. 4:**
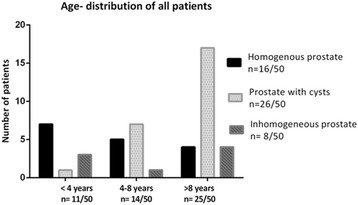
Presentation of the age-distribution of all patients, classified according to their morphological properties

**Fig. 5:**
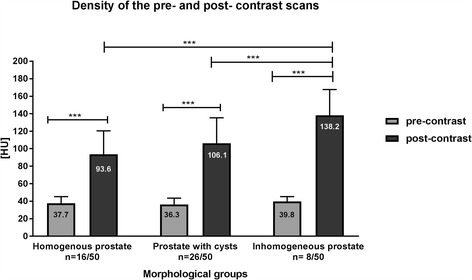
Attenuation values of the pre- and post- contrast scans of the prostate Statistically significant differences are indicated with * (p < 0.05), ** (p < 0.01) and *** (p < 0.001)

**Fig. 6:**
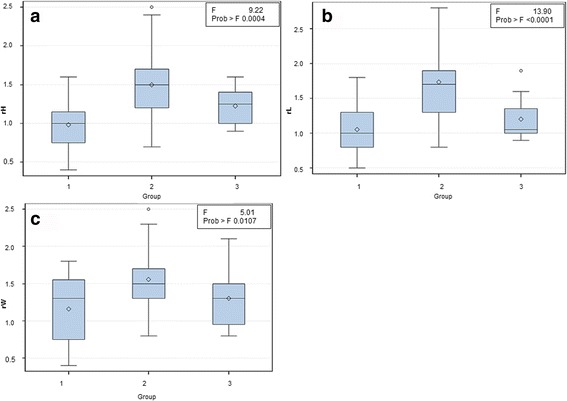
One-way analysis of variance between groups 1, 2 and 3 of the parameters: a) rH (ratio of the height) b) rL (ratio of the length). c) rW (ratio of the width). Prostatic size was measured in cm

Similar to the study of Lee et al. [15] the ratios of the prostatic size to the sixth lumbar vertebra were calculated. Accordingly, the relationship between the prostatic height, length and width to the length of the sixth lumbar vertebral body was calculated. The ratio describes the dimension of the prostate and displays alterations to the normal healthy prostatic size [15].

The attenuation values of the prostate were determined by measuring the Hounsfield Unit (HU) in predefined areas of the organ in transverse plane. Therefore, ellipsoid-shaped ROIs were drawn separately in each lobe of the prostate in pre- and post-contrast images (Fig. 2). The ROI’s size in the right and left lobe were uniform in size so that the ROI filled the entire lobes of the prostate. Mean and median HUs as well as minimum and maximum HU and the respective standard deviations were measured in every transverse slice over the whole length of the prostate. For each patient a median value of HUs of the whole prostate gland in the pre- and post- contrast scan was determined.

In dogs with prostate cysts the surrounding prostate tissue was further characterized by measuring its attenuation value. Therefore ROIs were placed in tissue with no visible cysts and HU were measured.

### Classification of the patients

The dogs were divided into three groups according to the dog’s age: Group A included dogs under 4 years; group B had dogs from 4 to 8 years, group C consisted of dogs over 8 years. Additionally, the study sample was likewise divided into three groups according to the appearance of the prostate gland on CT-images: group 1 dogs with homogeneous prostate tissue, group 2 patients with prostate cysts (diameter > 1.2 mm) and group 3 dogs with an inhomogeneous prostate tissue but without cysts﻿﻿ (Fig. 3)﻿. Furthermore, the patients with cysts were divided according to their cysts size in dogs with cysts smaller than 1 cm and larger than 1 cm. They were classified according to the visualization of the contrast and non-contrast CT images by one investigator.

### Sonographic examination

Additional data was available for most of the dogs from ultrasound examinations (n = 47/50) of the prostate gland. The ultrasound was performed at the Small Animal Clinic, University of Veterinary Medicine Hannover, Foundation, Germany. The ultrasound examination was performed with a Logiq 7 GE Healthcare ultrasound device (Wauwatosa, USA) using the B- Mode and a high frequency (8MHZ) curvilinear probe. Dogs were examined in dorsal recumbency. The prostatic size, echogenicity and tissue were evaluated during the ultrasound examination, but only the result of the tissue texture was analyzed in the present study.

### Statistical analysis

Statistical analysis was performed using the SAS® Enterprise Guide® 7.1 (Statistical Analysis Software, Heidelberg, Germany). For analyzing the differences between the median values of the three groups, a paired t-test was used (Ryan Einot Gabriel Welsch multiple range test). A p-value less than 0.05 was considered to be statistically significant.

To be able to evaluate the statistically significant differences between the groups and between pre- and post-contrast scans a factorial analysis of variance was performed.

The relation between the prostatic dimensions, age and the affiliation to the groups was analyzed by a Ryan-Einot-Gabriel-Welsch multiple range-test. The correlations of the prostatic dimension within the groups were analyzed by a Pearson Correlation test.

## Results

In the present study all dogs were divided into three age-groups and also into three morphological groups. In the age-group A (<4 years) (n = 11/50) with a mean age of 1.7 years most of the dogs had a homogenous prostate (n = 7/11). In the group B (4-8 years) (n = 15/50) with a mean age of 6.3 years some dogs had a homogenous prostate (n = 5/15) and some a prostate with cysts (n = 8/15) (Fig. 4).

The cyst’s size varied between 1.2 mm and 3.7 cm. Concerning the prostatic cyst’s size patients were divided in dogs with a cysts smaller than 1 cm (19/26) and larger than 1 cm (7/26). No statistically significant differences were found regarding the dog’s age between those two groups.

In the oldest group (>8 years) (n = 25/50) with a mean age of 10.7 years, prostates with cysts (17/25) were seen predominantly, while some dogs showed only an inhomogeneous prostate gland (n = 4/25).

Concerning the morphological groups, patients in group 1 (homogenous prostate) had a mean age of 5.2 years (between 0.7 years and 12 years), group 2 (prostate with cysts) =9.2 years (between 1.8 years and 13.8 years) and group 3 (inhomogeneous prostate) =6.4 years (between 1.3 years and 11.7).

The average age in the group differed statistically significantly (p < 0.05) between groups 1 and 2 and between group 3 and 2.

### Density values of prostatic scans

The density values measured in scans of homogenous (group 1), cystic (group 2) and inhomogeneous prostates (group 3) of each group are shown in Fig. 5. Comparing the median values of Hounsfield-Unit measurements in the right and left lobe of the prostate gland of each dog, there was no statistically significant difference (p > 0.05) between the lobes. Therefore, the detected values for the right and left prostate lobe were averaged for the further statistical evaluation.

For group 1, a mean density value of 37.7 HU (SD +/− 7.6) was achieved in pre-contrast scans and an attenuation value of 93.6 (SD +/− 26.8) in post- contrast CT-scans. In group 2, the pre-contrast mean density value was 36.3 (SD +/− 7.2) and the post-contrast density value was 106.1 HU (SD+/− 29.2). Furthermore, the mean density value of the prostate in the pre-contrast scans was 39.8 HU (SD +/− 5.5) and in post-contrast 138.23 HU (SD +/− 29.4) in group 3.

The tissue surrounding the cysts in group 2 had a mean density value of 40.4 HU (SD +/− 6.6) in pre-contrast scans and in post-contrast scans a mean density value of 121.7 HU (SD+/− 31.4).

Measurement of HUs in pre- and post-contrast scans showed statistically significant differences in the factorial analysis of variance (p < 0.05) in all groups.

A comparison of the attenuation in pre-contrast scans between all three groups showed no statistically significant differences. Comparing the median density of post-contrast scans, statistically significant differences were found between group 1 and group 3 (p < 0.0001) and group 2 and 3 (p < 0.0001). Between group 1 and 2 (p = 0.04) no statistical differences in measurement of HUs were found.

### Prostatic size

Concerning the prostatic size, average values varied between the three morphological groups (Table 2). The height of the prostate gland ranged between 1.1- 6.9 cm (mean 3.5 cm). In group 1 (homogenous prostate) the mean value of the height of the prostate amounted to 2.6 cm (ranging from 1.1 to 4.2 cm). The mean height in group 2 (prostate with cysts) was 4.0 cm (ranging from 2 to 6.9 cm). Group 3 (inhomogeneous prostate) had a mean prostate height of 3.2 cm (ranging from 1.7 cm to 4.6 cm).

**Table 2:** Prostatic characteristic results of the three morphological groups

Morphological Group	Height (cm)	Width (cm)	Length (cm)	Length of 6th LV	rH	rW	rL	Age (years)
1 (homogenous prostate)	2.6	3.1	2.6	2.6	1,0	1.2	1,1	5.2
2 (prostate with cysts)	4	4.1	4.7	2.7	1.5	1.6	1.7	9.2
3 (inhomogeneous prostate)	3.2	3.4	3.1	2.7	1.2	1.3	1.2	6.4

rH, ratio of the height, rW, ratio of the width, rL, ratio of the length, LV, lumbar vertebra

The mean width of all prostates from the different groups was 3.7 cm. The mean value of the width of the prostate in group 1 amounted to 3.1 cm (ranging from 0.9 to 4.4 cm). In group 2 the mean prostatic width was 4.1 cm (ranging from 2 to 8 cm) and in group 3 the mean value was 3.4 cm (ranging from 1.5 to 5.1 cm).

The mean length value of all prostates was 3.8 cm. The mean value of the prostatic length in group 1 was 2.6 cm (ranging from 1.3 to 4.3 cm) and in group 2 4.7 cm (ranging from 1.7 to 8.8 cm). Group 3 showed a mean value of the prostatic length of 3.1 cm (ranging from 1.6 and 5 cm).

Group 1 had the lowest ratio values. In group 1 the mean values for the prostatic rH was 1.0, for the rW 1.2 and for the rL 1.1. Due to the larger size of the prostates in group 2 rH and rL had significantly higher values than in group 1, whereas rW did not differ statistically significantly between the groups. In group 2 the mean values for rH, rW and rL were as follows: 1.5, 1.6 and 1.7, respectively. The mean values for prostatic ratios in group 3 were rH = 1.2, rW = 1.3, and rL = 1.2, respectively.

The correlation between the different parameters of the prostatic size (rH, rW, rL) was analyzed within the groups. Within all groups an overall good (> 0.6) and statistically significant (p < 0.05) correlation between rH, rW and rL was found (Table 3). The ratio of the height (rH) differed statistically significantly between groups1 and 2. The ratio of the width did not differ statistically significantly between the groups. On comparing groups 2 and 3 and also groups 1 and 2 the prostate length ratio differed statistically significantly﻿ (Fig. 6).

**Table 3:** Pearson’s correlation between the prostatic size parameters (rH, rW, rL) within the groups

	rH1	rH2	rH3
rW1	r = 0.77, p = 0.0005		
rW2		r = 0.5, p = 0.009	
rW3			r = 0.87, p = 0.005
rL1	r = 0.6, p = 0.02		
rL2		r = 0.8, p = 0.0001	
rL3			r = 0.85, p = 0.007

rH1,2,3, ratio of the height of groups 1;2,3; rW1,2,3, ratio of the width of groups 1,2,3; rL1,2,3, ratio of the length of groups 1,2,3

### Sonographic examinations

Furthermore ultrasound examinations were performed in 47 out of 50 patients. The results of the sonographic examination of each dog were compared with the results of pre- and post- contrast CT scan (Table 3). Dividing the patients in groups based on their appearance in CT imaging, 15 of the 16 patients of group1, 24 of 26 dogs of group 2 and all patients of group 3 underwent sonographic examination.

When no ultrasound results were available, patients were marked with two lines (--) in the ultrasound column.

In most cases (41/47) the ultrasonographic and CT examination came to the same description of prostate tissue structure (Table 4). However in six dogs post- contrast CT scans differed from the results of the sonographic examination as well as from pre- contrast CT scans. Five of those dogs showed an inhomogeneous tissue pattern in post- contrast, while pre-contrast CT and the sonographic examination showed a homogenous tissue. In one dog post- contrast CT revealed cysts in the prostate tissue, while pre- contrast and in the ultrasound images the prostate was homogenous as well.

**Table 4:** Results of the ultrasound examination of the prostate in comparison with the results of the CT scan images

Patient	Ultrasono-graphy	Precontrast CT scan	Postcontrast CT scan	Morphological group
#1	H	H	H	1
#2	H	H	H	1
#3	iC	iC, H	iC, H	2
#4	iC, pC, I	iC,pC, C	iC,pC, C	2
#5	H	H	H	1
#6	H	H	H	1
#7	--	I	I, iC	2
#8	H	H	H	1
#9	iC, I	I	iC, I	2
#10	H	H	H	1
#11	H	H	I, iC	2
#12	H	H	H	1
#13	H	H	I	3
#14	iC	iC, C	iC,C	2
#15	H	H	H	1
#16	H	H	H	1
#17	iC, I	iC, I, C	iC, I,C	2
#18	iC	iC, I	iC, I	2
#19	--	iC, I	iC, I	2
#20	iC, pC, I, C	iC, pC, I, C	iC, pC, I, C	2
#21	iC	iC, I	iC, I	2
#22	H	H	H	1
#23	I	I	I	3
#24	I	I	I	3
#25	iC, pC	iC, pC, I	iC, pC, I	2
#26	iC, I	iC, I	iC, I	2
#27	H	H	H	1
#28	iC,pC	iC, pC, I	iC, pC, I	2
#29	H	H	H	1
#30	iC	iC	iC	2
#31	iC, H	I	iC, I	2
#32	H	H	I	3
#33	iC, pC, C	iC, pC, C, I	iC, pC, C, I	2
#34	iC, C, I	C, I	iC, C, I	2
#35	iC, H	iC, pC, H	iC, pC, I	2
#36	iC, I	iC, H	iC, I	2
#37	H	H	H	1
#38	H	H	H	1
#39	iC	iC, pC, I	iC, pC, I	2
#40	iC, H	iC, I	iC, I	2
#41	H	H	H	1
#42	iC, I	iC, H	iC, I	2
#43	H	H	I	3
#44	iC, I	iC, I	iC, I	2
#45	H	H	I	3
#46	iC, pC, I	iC, pC, I	iC, pC, I	2
#47	H	H	I	3
#48	iC, pC, H	iC, pC, I	iC, pC, I	2
#49	I	I	I	3
#50	--	H	H	1

iC, intraprostatic Cysts

pC, paraprostatic Cysts

C, Calcifications

H, Homogenous prostate tissue

I, Inhomogeneous prostate tissue

## Discussion

A specific diagnosis of prostatic disorders is important for choosing the best therapy for the patients and for setting a realistic prognosis [8]. The most important tool for evaluating the prostate’s structure is the transabdominal ultrasound, which is partial limited by the intrapelvic position of the canine prostate gland.

Lee et al. [15] and Pasikowska et al. [18] used CT for evaluating the canine prostate gland. Both research groups came to the conclusion that CT is a beneficial tool for evaluating the morphology of the prostate.

The aim of the present study was to evaluate morphology of the canine prostate gland and to classify changes in relation to the patient’s age. Several studies evaluated the age-related changes of the prostate in dogs [19–21], but CT-changes in prostate texture have not been discussed corresponding to the dog’s age.

O′Shea [20] described the prostatic development in three phases: the first phase (1 to 5 years) defines the prostate morphology in a young adult dog as the physiological condition, in the second phase the prostate shows a hyperplastic growth (6 to 10 years) and the last phase is characterized by the senile involution of the prostate (11 years and over). These age-dependent changes in the prostate structure were also seen in the CT-examinations in the present study; young dogs mainly showed a homogenous prostate gland and only one dog had cysts in the prostate. Lowseth et al. [19] came to the same conclusion by means of histopathological investigations; dogs aged three years and younger were all classified as normal and none of these dogs had hyperplastic alterations.

With increasing age cystic alterations in the prostate gland increased in the present study. In the -age-group B (4-8 years), eight of fifteen dogs having cysts in their prostate. Similar results were reported for the second (hyperplastic) phase of O′Shea’s classification. Lowseth et al. mentioned that dogs aged six years and older showed evidence of complex BPH [19]. Patients in the present study aged six years and older mostly had a bigger inhomogeneous prostate or a prostate with cysts, findings which are indicative of a BPH and a progressive aging process. The prostatic ratio of the size was in the prostate with alterations bigger than in dogs with a normal prostate. One limitation of the present study was the assumption that no further examinations were made to confirm BPH. Relevant signs were these slightly increase in prostates size ratios and structural changes in tissue morphology. In further studies a calculation of the volume of dogs from the morphological groups could yield more detailed information about the prostatic size. Discussing age-dependent changes, one must point out that a definition of “age” or “geriatric life span” in dogs is difficult to find. In the present study the oldest age group of dogs included dogs with an age of over 8 years. This might be a limitation of the study. Since the aging process is depending on the adult body size, nutritional status and breed of the dog [22], this cut-off point may not be suitable for small breeds. Bellows et al. classified dogs as senior when they have a weight under 22.7 kg and have an age between 7-10 years, whereas dogs over 22.7 kg and with an age between 6-8 years [22]. Although the average age of dogs in the oldest age group in this study was with 10.7 years higher than 8 years, one has to keep in mind that some of these patients may still be mature but not geriatric. This may explain that in some dogs cystic changes were found. Further studies will be needed to describe changes of the prostate in CT examination in geriatric dogs, which take also into account the dog’s breed. Furthermore, the prostatic size in relation to dog’s weight should also be evaluated.

In the studies of Lee et al. and Pasikowska et al. a correlation between age and prostatic dimension was found but the effect of age on the morphological characteristics was not investigated with regard to detailed age categories. However, Lee et al. [15] described an irregular shaped prostate morphology in older dogs and most of these dogs had cysts in their prostate. The present study clearly demonstrated that dogs can be grouped into three age- categories depending on the CT findings of the respective prostates. In particular, inhomogeneity was significantly increased in older dogs in comparison to the younger groups of investigated dogs.

### Density values

As the classification of a homogenous, inhomogeneous and cystic prostate pattern was the subjective impression of one observer, measuring Hounsfield Units was considered to be a more objective parameter to specifically evaluate prostate tissue density in CT images. By placing the ROIs within the whole prostate, homogeneity of the whole gland could be detected. When measuring the density with single circle ROIs like Pasikowska et al. did, it can be assumed that at least some alterations within the prostate tissue were excluded from the evaluation.

In the post-contrast scans homogenous prostates (group1) showed the lowest density in comparison to the other groups. Generally, the normal prostate is relatively homogenous and the tissue has a hypodense structure compared to the hyperdense rectal wall [23]. Similar results were found in the present study and consequently the patients in group 1 can be assumed had healthy prostates.

The density values of the prostate with cysts in the post-contrast scans were higher than in group 1 but lower than in group 3. The post-contrast attenuation values of prostatic tissue surrounding cysts were higher than the ones of the homogenous and the total prostate with cysts. The study of Huggins et al. examined normal and hyperplastic canine prostates with histological analyses [24]. One conclusion of their study was, that hyperplastic prostate glands had more dilated vessels in the prostatic tissue compared to normal prostates [24]. The higher CT attenuation values in the post- contrast scans in the present study, can be indicates for a higher amount of vessels in the adjacent gland tissue.

The cyst itself is a hypodense structure [25] and accumulates no contrast agent [26]. Presumably, the lower attenuation values found within the cysts are balanced out with the higher attenuation values of the surrounding tissue. Therefore, the attenuation values of group 2 are within the average for the three groups.

Group 3 consisted of dogs with inhomogeneous prostates. In this group the HUs were higher in the pre-contrast as well as on the post-contrast CT scans compared to the other groups. Furthermore, inhomogeneity can be provoked by the aging process or by a heterogeneous blood distribution within the gland [15]. During the aging process the prostate can be hyperplastic and the histological pattern becomes irregular [20]. In older dogs the glandular tissue increases and the stromal tissue decreases in the prostate [27]. Consequently, these irregular patterns may cause a diffuse and inhomogeneous accumulation of contrast agent as can be seen in group 3. Schwarz et al. described the prostate tissue of a benign hyperplasia (BPH) in the CT as a heterogeneous parenchyma compared to the healthy gland [28]. Similar findings were described by Pasikowska et al. [18] who found that 75% of the patients with BPH had an inhomogeneous prostate and a mean age of 9 years. Additionally, cysts were often associated with the BPH [29].

Consequently, our findings for the prostates of dogs in group 2 and 3 indicate that some dogs may have a BPH which is represented by the altered prostatic tissue. In group 2 the prostate had cystic pattern and patients had a mean age of 9.2 years, moreover the inhomogeneous prostate of group 3 had a mean age of 6.4 years. One might conclude that both groups had a BPH. We can assume that, the BPH of dogs in group 3 was not as progressed the BPH of dogs in group 2. It can be hypothesized, that in the prostate of group3 with benign hyperplasia the cell division is higher than in the other groups which might also lead to a higher contrast enhancement.

### Sonographic examination

The results of the CT image evaluation and the ultrasound examination were compared. Especially in group 3 (inhomogeneous prostate) differences between the diagnostic imaging modalities were found. Five of the eight dogs had an inhomogeneous prostate in the post-contras CT-scan, but were homogenous in the ultrasound examination and in the pre-contrast CT-scans. This showed that especially due to the application of contrast agent in the CT, alterations are more visible and the CT images can provide additional information regarding the vascular system.

### Prostatic size

The prostatic size based on ultrasonography and x-rays was evaluated in several previous studies [10, 30–33]. Only two studies determined the prostatic size with the help of the CT [15, 18]. In contrast to the study of Lee et al., the present study and that of Pasikowska et al. devided the patients in groups.

As in the study of Lee et al. [15] and Pasikowska et al. [18] the ratios of the prostatic height, length and width to the sixth lumbar vertebral body were determined in this study. The ratio showed that prostates with alterations (cysts and inhomogeneous prostatic pattern) had higher ratio values on average than healthy homogenous prostates. Accordingly, the prostatic size increased due to cysts and aging processes as a consequence of age-dependent hyperplasia [1]. These results agree with the study of Pasikowska et al. that found a larger prostate size s in dogs with BPH than in healthy dogs.

The prostatic size ratios in the study of Pasikowska et al. were higher than in the present study. The healthy group of Pasikowska et al. had a higher mean age (mean 7.7 years) than the dogs with homogenous prostates in the present study (mean 5.2 years). This age difference could be an explanation for the different sizes of the prostates in the two studies.

### Limitations

The prostate completely encircles the urethra and our ROI shape covered the whole gland including the urethra. This could be a potential source of error. However, the urethra was included in all measurements, resulting in an almost equal influence on the measurement results of all groups.

As another limitation it has to be mentioned that due to the retrospective character of the present study older dogs were more often examined in the CT than younger ones. To study the age-dependent effects on the prostatic dimensions a higher number of subjects in the younger group would have been desirable.

## Conclusions

Our hypothesis that measuring the HUs of the prostate tissue in dogs can be helpful in diagnosing prostatic disorders could be confirmed. Pathological changes were reflected in the density values. The present study underlines that the density values of the canine prostate differed between the three morphological groups. Using contrast agent the significance of the evaluation results increased and may have the result that earlier prostatic changes could be visible than in the ultrasound examination. By measuring the density, homogeneity, the tissue surrounding prostatic cysts and the prostatic size it is possible to draw conclusions concerning the health of the prostate. Moreover, the study demonstrated that during age alterations increased and were visible in CT images.

## Abbreviations

CT: Computed tomography
BPH: Bening Prostatic Hyperplasia
